# Supplementary material for: Are carbon emissions trading and green financial instruments synergistic? -Comprehensive quantitative research based on content analysis
Source: PLoS One. 2024 Mar 7;19(3):e0298601. doi: 10.1371/journal.pone.0298601 (PMC10919601; doi:10.1371/journal.pone.0298601)
Supplement: S1 Data — (ZIP) [file pone.0298601.s001.zip › Data/地级市Z_F工作报告.zip!/░▓╗╒/╦▐╓▌╩╨/└┤╫╘╣1⁄2╓┌║┼í╛┬φ┐╦╩2╛▌═°í┐_2015.docx]

2015年1月19日在宿州市第四届人民代表大会第四次会议上

市长  史 翔

各位代表：

现在，我代表市人民政府，向大会报告政府工作，请予审议，并请市政协委员和其他列席人员提出意见。

一、攻坚克难，经济社会发展实现新突破

过去的一年，全市上下在省委、省政府和市委的正确领导下，深入贯彻落实党的十八大和十八届三中、四中全会精神，扎实做好稳增长、促改革、调结构、惠民生的各项工作，较好地完成了市四届人大三次会议确定的主要目标任务。

初步核算全市生产总值1126亿元，增长9.7%；财政收入114.3亿元，增长14%；社会消费品零售总额344亿元，增长13.6%；城镇、农村常住居民人均可支配收入21775元和8147元，分别增长9%、13%；节能减排达到省控目标，动态指标继续保持全省前列，发展质量效益同步提升。荣获2013年度省政府目标管理绩效考核先进单位和经济发展先进奖。

一是工业发展量增质升。坚持工业、招商、项目、园区四位一体推进，工业总量加速扩张，质量明显提升。规模以上工业增加值336亿元，增长11.8%。规模以上工业企业1010户，产值超亿元企业440户。投入持续加大，工业投资532.2亿元，增长22.1%。规模以上工业主营业务收入增长20%，实现利润、利税分别增长50%、30%。工业用电量增长5.4%。转型升级步伐加快，高新技术产业产值突破130亿元。大力实施“3111”工程，各县区、园区实现首位产业产值292.9亿元，增长24.8%，实现首位产业税收8.2亿元，增长23.8%，顺利完成工业项目百日攻坚三个“65”的目标任务。四大园区加快发展，市经开区新引进上市公司3家，安特生化二期等退城进园项目相继建成；鞋城获中国现代制鞋产业城称号，野力体育等34家企业投产；宿马园区获首批中国青年创业示范园区称号，入驻项目86个；市高新区建设提速，华为等一批数量可观的现代通信及应用高新企业相继落户。

二是有效投入持续加大。项目建设提质提效，固定资产投资945.8亿元，增长22.3%。超额完成省“861”项目建设任务。完善项目调度推进机制，621个亿元以上项目加快建设，冠军建材一期、国口酒业、鸿丰生物、麒翔林产品等110个亿元以上项目建成投产，186个重点项目完成投资324亿元。宿淮铁路、徐明高速正式通车，济祁高速宿州段主体工程完工，郑徐客运专线、符夹线扩能改造加快推进，宿淮铁路蒿沟站动工，国省干线公路改造完工120公里。聚力强攻招商引资，与上海张江高新区正式签订战略合作框架协议，实际利用内资1350亿元，利用外资5.4亿美元。深化与央企、省企、知名民企合作，引资283.5亿元。

三是城乡面貌显著改观。全市实施重点工程213个，完成投资220亿元。规划工作继续加强，主城区基本实现控制性详细规划全覆盖，四个县城总体规划修编全部启动。重点工程亮点纷呈，市文化艺术中心、汴北水厂建成运行，市规划馆、体育馆加快建设，万达广场、元一新天地顺利推进。道路通达能力提升，宿城大外环75%路段建成，环内面积超200平方公里，南北环公跨铁立交桥、宿州大道新汴河大桥建成通车，“六纵十横”路网形成。启动国家园林城市创建工作，新增绿地586万平方米，新建绿道47.7公里，新汴河景观工程成效初显。征迁拆违步入良性发展轨道，控违工作成效显著。荣获省级文明城市称号，埇桥、灵璧进入省级文明城区、文明县城行列，砀山、泗县进入创建先进行列。县镇发展提速，“555”工程扎实推进，四个县开工建设重点项目79个，“五个一”工程进展顺利；符离等8个镇被评为全国重点镇；萧县、埇桥被评为全省美好乡村先进县区，第二批37个示范村建设稳步推进。“三线三边”环境整治成为宿州的响亮品牌，3座垃圾焚烧发电厂开工建设。

四是现代农业提速升级。粮食总产79.3亿斤，实现“十一连丰”，小麦单产再创新高。扎实推进现代农业“两区”建设，20个示范基地及园区完成投资7.9亿元，产值92.6亿元。积极培育现代农业产业联合体等新型农业经营主体，农民专业合作社数量居全省首位。农产品加工产值突破900亿元，市级以上龙头企业发展到438家，农业产业化综合水平保持全省第一方阵。农田水利三年提升工程超额完成，新增高效节水灌溉面积22万亩，埇桥区蝉联“江淮杯”一等奖，新汴河治理、淮水北调、小农水重点县等重点工程加快建设。实施精准扶贫，新增脱贫人口10万人。深入推进森林增长工程，新造林14.7万亩。林业总产值突破350亿元，埇桥获全国唯一的杨木产业示范区称号。秸秆禁烧和综合利用工作成效显著。严守耕地保护红线，连续六年实现占补平衡，工矿废弃地复垦利用列入国家试点。

五是改革活力不断释放。通过简政放权，深化行政审批制度改革，市级行政审批事项减少50.6%。全面落实注册资本认缴登记制，新登记注册企业8354户，增长150%。土地承包经营权确权登记颁证试点工作进展顺利，新增土地流转面积88.5万亩，农村集体产权制度改革逐步推进。深化财税体制改革，县级以上政府及部门预决算全部公开。县级工商、质监、食品药品监管机构职责实现“三合一”。12家县级公立医院综合改革完成。实施金融“五个一”工程，出台金融支持工业经济加快发展“十二条”措施，贷款增速居全省前列。全面完成农村合作金融机构改制任务，清收不良贷款11亿元。交通银行宿州分行开业，新增各类银行网点11个。市中小企业融资担保公司市场化改革初见成效。亿海矿山在新三板实现上市“零”突破，3家企业在省股权托管交易中心成功挂牌。砀山酥梨在天津渤海商品交易所成功上市。创新交通商贸物流管理服务机制，入选粤苏皖赣四省物流大通道主要驿站及甩挂中心城市，被列为全省唯一的交通运输物流试点市。

六是民计民生有效改善。全市民生支出206亿元，占财政总支出的83.6%。33项民生工程全面完成，投入资金70.6亿元，增长15.8%。10件惠民实事顺利实施，新建、改扩建、增设附属幼儿园100所，投放52辆城市公交车、3000辆公共自行车，市120指挥中心建成使用，科技馆、妇女儿童活动中心即将开工，市妇幼保健院建设加快推进。“八小水利工程”顺利实施，解决66万人饮水安全问题。新建各类保障房3.2万套，开工率、基本建成率均居全省前列。城镇登记失业率控制在3.6%以内。“五险”参保185.4万人次。基本实现医疗保险省内异地就医双向结算，新农合大病保险实现县域全覆盖。渔民上岸安居工程顺利完成。

七是社会事业全面发展。完成义务教育学校标准化建设180所，皖北卫生职业学院批准成立。加强公民思想道德建设，入选“中国好人”4名、“安徽好人”8名。隋唐大运河申遗成功，新增2项国家级非物质文化遗产，砀山唢呐载誉美国第十届亚裔节。举办第三届WTA国际女子网球公开赛、首届全民健身运动会，在省运会勇夺55枚金牌。磬云山获批国家级地质公园，新增4A级景区2家。举办第二十届亚洲旅游业金旅奖峰会。落实“单独两孩”生育政策，计生工作跨入全省前列。空气环境质量控制目标完成较好，新增1个省级生态乡镇、9个省级生态村。社会治理重心下移，“平安宿州”建设扎实推进，社会服务管理信息化加快建设。安全生产形势总体平稳，超限超载势头有效遏制，应急管理体系不断完善，食品药品监管规范有序，信访秩序稳步向好。第三次全国经济普查取得阶段性成果。荣获全国红十字会系统先进集体、防震减灾工作先进单位。砀山县获全国民族团结进步模范集体。国防动员、人民防空和双拥工作成绩显著，宿州舰即将入列。民族宗教、外事侨务、物价、对台、气象、妇女儿童、残疾人、老龄、烟草等工作取得新成绩。

各位代表，我们把振兴县域经济作为全市发展的主攻方向，吹响了振兴县域经济的冲锋号，短短一年就初见成效。县域生产总值、规模以上工业增加值、战略性新兴产业产值等指标占全市比重明显提升。工业增加值过亿元、财政收入过千万元乡镇分别达到55个和49个。这充分证明市委、市政府的决策是完全正确的，也坚定了我们进一步发展县域经济的信心和决心！

过去的一年，我们高度重视政府自身建设。扎实开展党的群众路线教育实践活动，认真落实中央八项规定、国务院“约法三章”和省市“三十条”要求，恪守“三严三实”，切实解决“四风”问题，“三公经费”下降25.3%。持续推进“点燃激情、提升效能”等活动，有效整治“庸懒散奢”、“中梗阻”和“基层板结”现象。完善市政府工作规则，规范政府举债行为，率先公布权力清单、责任清单，严格执行重大决策和规范性文件合法性审查制度，政府系统重大事项决策行为进一步规范。坚持领导干部工作计划、工作动态网上公开，积极推进网上审批、网络问政、微博问政，政府工作透明度不断提高。自觉接受人大及其常委会监督、政协民主监督和社会舆论监督，办理人大代表议案和建议96件，政协委员提案256件。强化行政监察和审计监督，勤政廉政建设取得新成效。

各位代表，一年来的奋斗历程，让我们深切体会到：必须坚持整体承接、双轮驱动、三区共进、四化同步，一张好的蓝图绘到底，才能在未来发展中久久为功、不折腾；必须坚持抓主抓重、苦干实干、提速提效、争先争优，营造干事创业的好氛围，才能不断超越自我、不懈怠；必须坚持工业强市兴县，持续扩大有效投入，才能在区域竞争中奋力崛起、不走偏；必须坚持振兴县域经济，叫响工业“3111”和城乡建设“555”两个品牌，打基础、补短板、增后劲，才能让整体经济稳扎稳打、不空虚；必须坚持改善民生，让全市人民生活得更加幸福、更加殷实，才能赢得人民群众的真心拥护、不失望。

奋斗历尽艰辛，成绩来之不易。这是市委总揽全局、坚强领导的结果，是市人大、市政协和社会各界监督支持的结果，更是全市上下同心协力、团结拼搏的结果。在此，我代表市人民政府，向奋战在各行各业的全体劳动者，向离退休老同志，向各民主党派、工商联和人民团体，向中央和省驻宿单位、人民解放军驻宿部队、武警官兵、公安干警，向参与宿州建设的海内外客商、企业家、广大援宿干部，以及支持宿州发展的各界人士，表示衷心的感谢！

回顾过去的一年，我们也清醒地认识到，宏观经济环境发生了深刻而根本性的变化，经济发展步入了新常态，结构调整阵痛加剧，资源要素约束凸显，发展面临着全新的挑战。反映到我市经济社会发展的微观层面，与全国、全省一样，经济下行压力加大，经济运行中存在诸多困难和问题。经济总量不大，县域经济不强，新的增长动力不足；工业基础薄弱，企业生产经营转型不适，战略性新兴产业规模偏小，工业强市兴县之路任重道远；财政增收放缓，政府债务风险开始显现，社会保障和公共服务水平与群众期盼还有不小差距；政府职能转变远不到位，作风不实、服务不优、效能不高等问题仍不同程度存在；少数干部担当意识不强，重形式轻实效、重布置轻落实等现象仍较突出。对此，我们一定高度重视，认真解决。

二、坚定信心，奋力打造经济社会发展升级版

2015年是全面深化改革的关键之年，是全面推进依法治市的开局之年，也是全面完成“十二五”规划的收官之年，做好今年的政府工作意义十分重大而深远。当前，世界经济仍处深度调整期，国内经济发展进入速度变化、结构优化、动力转换的新常态，我市正处于爬坡过坎、攻坚转型的关键时期，发展任务艰巨繁重。同时，我们充分认识到，我国经济总体向好的基本面没有改变，全省仍处于重要战略机遇期，我市已经进入新型工业化、城镇化的加快发展阶段，随着“一带一路”、长江经济带、中部崛起、中原经济区、皖北振兴等政策红利的不断释放，全市发展的韧性、潜力和空间巨大。我们要深刻认识新常态、主动适应新常态、积极引领新常态，趋利避害、顺势而为，保持定力、迎难而上，努力开创宿州发展新局面。

今年政府工作的总体要求是：全面贯彻党的十八大、十八届三中、四中全会和中央、全省经济工作会议精神，坚持稳中求进工作总基调，主动适应经济发展新常态，以提质提效为中心，坚持依法治市和创新驱动，持续扩大有效投入，加快振兴县域经济，继续实施“全市工业和城乡建设三年振兴计划”，狠抓改革攻坚，强化民生保障，全面完成“十二五”目标任务，努力建设“实力宿州、活力宿州、和谐宿州、幸福宿州”。

经济社会发展主要预期目标是：全市生产总值增长9%左右，财政收入增长9%左右，固定资产投资增长15%左右，规模以上工业增加值增长12%左右，社会消费品零售总额增长13%左右，城镇、农村常住居民人均可支配收入分别增长9%和10%左右，城镇登记失业率控制在4.5%以内，“十二五”节能减排任务全面完成，主要动态指标继续位居全省前列。重点做好七个方面工作：

（一）主攻县域经济。坚持把振兴县域经济作为全市经济工作的重中之重，以“3111”工程为抓手，以“三个集中”为平台，持之以恒推动县域经济振兴。

培育首位产业。围绕“一年打基础，两年见成效，三年上台阶”的目标，深入实施“3111”工程，促进产业集聚集群发展，培育县域经济支柱。支持埇桥做大做强服装和板材产业，加快中国中部服装产业城建设；支持萧县壮大建材产业规模，加快张江萧县高科技园、陶瓷产业园建设；支持泗县培育壮大机电产业，高水平建设泗海工业园、泗县当涂现代产业园；支持灵璧积极发展新产业、砀山壮大果蔬食品产业，鼓励两县在食品产业上错位发展。实现县区首位产业产值360亿元、工业税收16亿元（其中首位产业税收9亿元），不断提升县域经济综合实力。

注重政策扶持。完善支持县域经济发展的政策措施，实行“一县一策”，主攻薄弱环节，在产业布局、招商引资、项目安排、土地指标、基础设施、政府债务、财政专项资金等方面向县区倾斜。充分利用土地倾斜政策，扩大城乡建设用地增减挂钩规模。实行要素跟着项目走，以项目定用地、以投资额定用地，合理配置土地指标，拓展县域经济发展用地空间。鼓励金融机构扩大县域信贷投放规模、下放业务权限，强化县域经济发展的金融支撑。

激发内生动力。建立权责对等的行政管理体制，把能放的权力放给基层，增强县区自主发展的主体意识。鼓励县区抢抓皖北振兴“新十条”政策机遇，用足用活省、市出台的扶持政策。创新县域经济考核方式，加大首位产业产值、工业税收、园区和城乡建设等指标考核奖惩权重。量化工作目标，坚持县域经济定期调度、首位产业专项调度，引导县区你追我赶、竞相发展。

（二）突出工业主导地位。坚持把工业强市兴县真正作为推动发展的第一要务，定向施策、精准发力，积极培育新增长点，以工业质量、效益的提升，带动整体经济的跃进。

强化招商引资工作。坚持“工业项目、招大引强、税源企业、环境保护”原则，创新招商引资方式，全市实际到位内资增长15%，实际利用外资增长10%。深入推进产业招商、精准招商、以商招商，制定首位产业招商规划，瞄准首位工业产业链和配套产业，招引“顶天立地”的首位工业项目，同时引进一批“铺天盖地”的优质工业项目。继续坚持招商周、领导带头招商、招商任务计划公开等制度，开展市直单位与园区“挂钩招商”活动。完善招商引资目标任务考核办法，加大新开工项目、竣工项目、首位产业项目、亩均投资强度和税收贡献的考核权重。依法合规地清理和规范税收等优惠政策，持续优化招商环境。

夯实园区发展平台。全面开展“园区项目建设年”活动，推动园区产城一体、融合发展。市经开区重点发展生化医药产业，争创国家级开发区。鞋城重点发展制鞋产业，更多引进整鞋、物流及配套项目，实现工业总产值70亿元。宿马园区重点发展现代食品产业，加快推进王老吉项目、黄池食品产业园建设，完成固定资产投资50亿元。市高新区争取正式获批，重点发展大数据和云计算产业，完成固定资产投资20亿元。四个园区固定资产投资增速不低于30%、税收增速不低于20%。推进各类园区转型升级，严格规范土地出让行为，积极盘活闲置和低效利用土地，新建多层标准化厂房10万平方米以上，不断提高土地节约集约利用率。

大力发展实体经济。持续扩张工业总量，全市规模以上工业增加值实现360亿元，工业投资完成600亿元。认真落实省、市促进经济持续健康发展“三十条”意见，加大“一企一策”帮扶力度，新增规模以上工业企业100户。实施创新驱动，出台战略性新兴产业发展意见和考核细则，力争战略性新兴产业产值增长19%左右。力促皖北药业、雪龙化纤等退城进园项目尽快投产达效，加快企业转型升级。在资金、政策、项目等方面给予重点扶持，培育一批“专精特新”中小企业。安排专项扶持资金，制定民营经济分类考核办法，加快民营企业发展，新增私营企业2000户、个体工商户10000户。

推动项目提速提效。坚持经济工作项目化、项目工作责任化、责任落实制度化，加大项目谋划、争取和实施力度，组织实施亿元以上和10亿元以上项目，固定资产投资突破1000亿元。严格落实“五个一”工作法，深入实施市级、县处级领导干部联系包扶项目制度，力促钱营孜低热值煤发电、阿尔法游戏产业园、红旗电缆、朱山光伏发电等尽快开工，确保亿帆药业、欣意电缆、瑞年健康产业园、罗盛达陶瓷、立诚包装等建成投产。力争360个项目列入省亿元以上重点项目投资计划，年度投资240亿元。认真谋划“十三五”规划项目，调整充实“5161”项目库，储备规模保持在万亿元以上。

（三）统筹推进城乡建设。深入推进以人为核心的新型城镇化，大力实施“城乡建设提升年”活动，实施176项重点工程，完成投资180亿元以上，城镇化率提高1.5个百分点以上。

突出中心城区建设。加强规划衔接，建立“阳光规划”制度，启动大宿城空间战略发展规划编制，理顺园区规划管理体制。坚持东向高铁、北跨汴河、南展新城、西越高速，强力推进五大片区建设，重点实施四大类工程，实现“双75”目标。加快路网建设，新建道路桥梁工程16个，确保大外环全线建成贯通，加快芒砀路等项目进度，建成人民路唐河桥，打通断头路。加快新汴河景观工程建设，开工建设植物公园、森林动物园，继续实施清水工程。

加强县、镇、村建设。按照“三治三增三提升”要求，以完善基础、配套功能、提升品位、打造特色为核心，继续实施“555”工程。在县城建设上，四个县各建设一座垃圾焚烧发电厂，新建一处不低于10万平方米的保障性安居工程，建设10个街头游园，建成一个符合标准的城建档案馆，新建一座日处理能力不低于2.5万吨的污水处理厂。在镇村发展上，县区各完成一个镇当县建的控制性详规编制，完善城乡环卫一体化、市场化管理体系，完成美好乡村示范村供排水和污水管网建设，新增10个以上集体经济收入过10万元的村，五个县区共新增土地流转面积70万亩以上。在工作要求上，四个县各完成土地出让收入10亿元，收储土地1000亩，征迁整理净地不低于500亩，完成县城总体规划修编完善工作，完成县城地下管网资料档案整理和存档备份。

完善城市功能。加快推进京沪高铁宿州东站扩建、宿淮铁路蒿沟站、郑徐客运专线等工程建设，确保济祁高速宿州段建成通车，构筑“三纵两横”高速公路网，实现县县通高速。强化与周边地市、县区的路网对接，加快打通跨区域断头路，逐步把宿州的区位优势转化为发展优势。继续推进宿灵泗、宿萧砀、206国道快速通道等一级公路建设，着力提高道路通畅水平。升级改造县乡公路150公里，实施村级公路网化工程200公里。力争市客运中心站主体工程完工。实施淮水北调沱河截污治理，加快汴北污水处理厂建设。开展“电网建设年”活动，建设宿州骨干网架。支持铁塔公司发展，推进智慧城市建设。大力推广政府与社会资本合作（PPP）模式，适时适量发行市政债，建立多元化、可持续的城镇化投融资新体制。

加强城市精细化管理。巩固省级文明城市创建成果，全面提升城市管理常态化、市场化、精细化水平。深入实施“五化”工程，突出抓好“七乱”整治，重点推进城市“五大节点”改造。争创国家级园林城市，让绿化“进机关、进学校、进社区、进小区、进公园、进企业”，增绿800万平方米。深入实施森林增长工程，扎实推进“三线三边”绿化提升和“四森”创建工作。加快城区雨污分流管网建设。加大公交投入，提高公共交通的吸引力和分担率。规范小区物业管理。完善大城管工作机制，积极推进环卫、市政体制改革，完善数字城管运行机制。

（四）大力发展现代农业。坚持把“三农”工作作为重要抓手，转变农业发展方式，夯实农业发展基础，加快推进农业现代化，持续增加农民收入。

努力转变农业发展方式。围绕现代农业“两区”建设，大胆改革创新，努力打造埇桥灰古核心示范区。加快构建现代农业经营组织体系，鼓励支持新型农业经营主体加快发展，培育市级以上示范家庭农场50个、示范合作社50个、新型职业农民2000人。加速推进农业产业化，由“生产导向”向“消费导向”转变，实现农产品加工产值1000亿元，市级以上龙头企业达500家。继续稳定粮食生产。实现林业总产值380亿元以上。实施新汴河治理、淮水北调干线及配水工程等重点水利工程，推进农田水利基本建设，解决63.2万人饮水安全问题，落实最严格的水资源管理制度。强化农业科技创新，促进农技、农机、农艺结合。推进农村土地制度和集体产权制度改革，稳妥开展宅基地管理、集体经营性建设用地入市改革试点。完成灵璧、萧县农村土地承包经营权确权登记颁证工作，加快乡镇土地经营权流转市场建设。

不断推进美好乡村建设。坚持“物的新农村”和“人的新农村”建设齐头并进，加快建设第二批37个美好乡村示范村。突出兴业富民，发展壮大农村集体经济。深入推进“三线三边”环境治理和秸秆禁烧工作，扩大秸秆还田面积，加快建设4座生物质能秸秆发电厂。

全力打好扶贫开发攻坚战。坚持“输血”和“造血”相结合，实施扶贫开发“五个一”重点工程，推进科学扶贫、精准扶贫，抓好产业扶贫、项目扶贫、智力扶贫，实现农村贫困人口稳定脱贫8.5万人，脱贫率达15.6%，贫困发生率降至6.6%。

（五）积极开拓现代服务业。坚持把发展壮大现代服务业作为重要的增长极，完善产业体系，推进业态创新。

加快培育现代服务业。大力发展云计算、大数据、物联网等新兴业态，积极推进亿赞普、中国网库等项目建设。出台加快电子商务发展的实施意见，推进电商、网购、快递融合发展。加快高新区省级电子商务产业园建设，培育招引一批标志性、引领性的电商企业。推动阿里巴巴宿州产业带运营，力争200家企业上线。抓住省交通运输物流试点市机遇，积极融入粤苏皖赣四省大通道和中原经济区五省大通道。完善交通商贸物流发展规划，大力发展第三方物流，建设智能物流信息平台，形成市、县、乡、村四级物流网络。

改造提升传统服务业。推进大型商业项目建设，开工建设苏宁广场，加快南翔恒泰、国购广场建设，争取百大农产品物流中心三期、创智物流园等投入运营。完成商业网点规划修编，提高现有商业设施利用率，推进商业商务中心人气集聚和业态繁荣。促进文化、旅游产业融合发展，全力打造奇石、书画、戏曲、马戏、唢呐等文化产业名片，推进南翔云集文化商业街等项目建设。抓好皇藏峪5A级景区创建，加快各县区旅游资源整合，力争旅游总收入超过80亿元。继续推进放心早餐、家政服务网点、社区便利店等便民服务设施建设，打造15分钟便民服务圈。新增2家省级服务业集聚区。

稳妥推动金融创新。继续实施金融“五个一”工程，严格执行金融机构支持地方经济发展考核制度，出台金融扶持“三农”发展办法，全年新增贷款120亿元。引进大中型股份制商业银行、村镇银行，鼓励农商行和村镇银行增资扩股，加快构建多层次金融服务体系。加强政策性担保体系建设，建立银政担风险分担机制。组建政策性小贷公司，实施“助保贷”计划，帮助解决中小企业贷款接续资金问题。加快企业上市步伐，重点推动4家企业在新三板上市。拓展直接融资渠道，推广表外融资、股权投资、债券融资等金融创新产品。加快推进信用体系建设，继续清收各类不良贷款，规范民间融资，持续优化金融生态环境。推动现有市级政府融资平台公司平稳转型发展。

（六）进一步深化改革开放。认真落实中央和省里全面深化改革的各项决策部署，不失时机推进各项重点改革，使改革举措有效转化为发展动力。

推进各项重点改革。坚持问题导向，突出重点领域，以经济体制改革为牵引，着力解决制约经济社会发展的关键问题。稳步推进国企改革，大力发展混合所有制经济。积极推进财税体制改革，完善全面规范、公开透明的预算管理制度。进一步加强政府债务管理，切实防范债务风险。完善职称评定制度改革。扩大“营改增”范围。扎实推进户籍制度改革。推动公务用车制度改革。全面推进公立医院综合改革，逐步破除以药补医。

开创对外开放新局面。扶持和培育实体企业扩大自营进出口，促进澳利安食品、皖神、东大木业、科技食品、新林纺织等做大做强，壮大东艺鞋业、力成生物等骨干企业，力争全市进出口额增长10%。提升贸易便利化水平，加快公用型保税仓库和海关监管、检验检疫查验场所建设，争取海关和出入境检验检疫机构正式开关开检。

（七）着力保障和改善民生。始终把群众利益放在首位，提高公共服务水平，更好满足人民需求，确保社会和谐稳定。

促进社会事业全面发展。全面改善义务教育薄弱学校基本办学条件，完成149所义务教育学校标准化建设。加强教师队伍建设，促进优秀教师轮岗任教基层边穷学校。保障进城务工人员随迁子女公平入学。加快宿城一中城南分校建设，建成宿马九年制公立学校。加快职教园区建设，支持职业技术学院转型发展，推进省鞋业人才培养基地和鞋业学院建设。培育诚信文化，启动荣誉市民评选。增加基本公共文化产品供给，积极推进县“两馆一场”建设，大力提升乡镇综合文化站、农村公益电影放映等文化惠民工程服务质量。加强大运河世界遗产保护利用工作。加快市立医院新区、市妇幼保健院建设，创建国家级慢性非传染性疾病综合防控示范区。落实好“单独两孩”政策，稳定适度低生育水平。广泛开展全民健身运动，举办市第五届运动会，基本建成市体育馆。实施城乡困难群众殡葬救助，推进公益性公墓建设。严格落实大气、水、土壤污染防治行动计划，推进水源地保护，加强环境空气质量监测能力建设，让宿州大地天常蓝、山长绿、水更清。加快推进采煤沉陷区综合治理，实施好工矿废弃地复垦利用项目，推进矿山综合整治。全面完成“六五”普法任务，加强国防动员、人防和民兵预备役建设，争创全国双拥模范城“七连冠”。扎实做好民族宗教、档案、地方志、残疾人、防震减灾、红十字会、外事侨务和对台工作，协调推进供销、气象、烟草等工作。

积极创新社会治理。健全安全生产“党政同责、一岗双责”责任体系，构建安全生产长效机制，严防重特大事故发生。加强道路交通、消防安全管理，继续保持治超高压态势。强化食品药品监管，完善从生产加工到流通消费的全程监管机制。推行“阳光信访”，加快建设网上信访信息平台，畅通民意表达渠道。完善应急管理机制，提升突发事件防范、应对和处置水平。创新社会治理方式，完成10个“四有”标准示范社区建设。全面推进社会服务管理信息化建设，实现村居全覆盖。深化“平安宿州”建设，加快建设立体化、数字化治安防控体系，依法加强网络社会管理，确保群众安居乐业、社会安定有序。

健全就业和社会保障体系。扎实推进创业型城市建设，落实并完善新一轮促进创业扶持政策，加大公共就业创业服务体系建设。认真组织开展“就业提升年”活动，统筹做好高校毕业生、城镇困难人员、下岗失业人员、退役军人等重点群体就业工作，城镇新增就业3万人。深入实施“十百千万”人才引进工程，加大创新创业人才引进培养力度，充分激发各类人才创造活力。深化收入分配制度改革，推进企业工资集体协商和集体合同制度，探索农民工工资按月打卡发放制度。积极做好各类灵活就业人员的参保工作，落实社会保险关系转移接续政策，加快社会保障一卡通建设。“五险”参保达到180万人次，城乡居民社会养老保险参保335万人以上。全面推进城镇职工和城镇居民大病保险工作，完善新农合报销政策。提高城乡低保和农村五保供养标准，规范低保、医疗救助和临时救助工作。加快发展养老服务业。健全农村留守儿童、妇女、老年人关爱帮扶体系，做好生活无着人员社会救助工作，保障孤儿基本生活。做好价格监测预警，稳定民生物价。新建保障性安居工程10个，建设保障房2.7万套，交付45万平方米。

各位代表，民生是为政之本。今年我们将深入实施33项民生工程，并集中力量再办10件符合民意、贴近民需、能解民困的惠民实事，让群众切身感受到民生在改善、福利在增加。

1．市区建设绿道10公里，新建一批街头游园

2．市政公厕全部免费开放，市区新建一批环保公厕

3．设置一批便民示范服务点，增设一批流动综合服务工作室，投放一批便民服务车辆

4．规范城区洗车点，设置一批洗车疏导场

5．市区新投放公共自行车1500辆，建设一批站点

6．全面发行宿州城市一卡通

7．完成市区中山街商品市场改造提升工程

8．新增60辆新型环保公交车，完成西北新区公交枢纽站和高新区公交首末站建设

9．建设食品药品示范街

10．新建一批垃圾转运站

三、依法施政，全面建设法治政府

依法治国、建设法治宿州，对政府工作提出了新的更高要求。我们必须强化政府自身建设，坚持在党的领导下、在法治轨道上开展工作，加快建设职能科学、权责法定、公开公正、廉洁高效的法治政府。

切实树立法治政府意识。坚持法定职责必须为、法无授权不可为，把“依法依规、集体决策、公开透明、终身负责”贯穿履职尽责全过程，确保在法治轨道上推进各项工作。时刻牢记法律红线不可逾越、法律底线不可触碰，养成遇事找法、办事依法、坚决守法的习惯。大力推动学法、尊法、用法、守法，善于运用法律手段管理经济和社会事务。

切实提高依法行政能力。坚持把公众参与、专家论证、风险评估、合法性审查、集体讨论决定作为重大决策法定程序，健全重大决策终身责任追究制度及责任倒查机制，切实提高决策质量。在全市推行政府法律顾问制度，所有政府决策必须于法有据。加大政务公开，及时回应社会关切。深化行政执法体制改革，探索建立执法全过程记录制度，实行重大执法决定法制审核制度，严禁违法执法、滥用自由裁量权、随意作为。

切实转变政府职能。减少对微观经济的干预，强化公共服务、市场监管、社会治理、环境保护等职责，努力让政府更好归位、市场更大发力、群众更多受益。把简政放权、放管结合推向深入，用政府权力“减法”换取市场活力“加法”和创新创业“乘法”。建立最严格的行政审批事项准入制度，继续推进“四个一”工程，防止行政审批事项边减边增、明减暗增，市本级审批事项再减少30%。公布运行市县政府权力和责任清单制度，探索建立负面清单制度。实施涉企收费清单制度，取消、归并41项收费项目，减少34%。

切实改进工作作风。巩固党的群众路线教育实践活动成果，将群众观点植根于思想中、落实到行动上，始终把维护和发展人民群众的根本利益作为最高追求。坚持“作风建设永远在路上，永远没有休止符”，深入推进“点燃激情、提升效能”活动，加大“四风”和“庸懒散奢”治理力度。坚持周通报、月调度、季总结工作制度，以抓铁有痕、踏石留印的劲头，埋头苦干抓落实。坚持在其位、谋其政，敢于负责、勇于担当，坚决克服懒政、怠政，坚决惩处失职、渎职，打通政策落实“最后一公里”，确保政令畅通、令行禁止。

切实强化监督制约。深入推进惩治和预防腐败体系建设，认真履行党风廉政建设主体责任，使广大干部明底线、知敬畏。自觉接受人大及其常委会监督和政协民主监督，切实办好人大代表议案、建议和政协委员提案，广泛听取人民团体意见，全面接受新闻媒体和人民群众监督。严格规范行政权力特别是腐败易发领域行政权力的运行，对公共资金、国有资产、国有资源和领导干部履行经济责任情况实行审计全覆盖。强化行政监察，严肃查处违纪违规行为。进一步落实中央八项规定和省市“三十条”要求，贯彻厉行节约反对浪费条例，严控“三公经费”支出。加强公务员队伍建设，所有公务员都要廉洁奉公，勤勉尽责，在严格律己中担当责任、在清风正气中干事创业，努力以自己的辛苦指数换取百姓的幸福指数。

各位代表！

面对发展的新常态、人民的新期待，我们深感责任重大、使命光荣。让我们紧密团结在以习近平同志为总书记的党中央周围，在市委的坚强领导下，团结拼搏，苦干实干，共同谱写中国梦的宿州新篇章！
